# Identification of Bipotential Blood Cell/Nephrocyte Progenitors in *Drosophila:* Another Route for Generating Blood Progenitors

**DOI:** 10.3389/fcell.2022.834720

**Published:** 2022-02-14

**Authors:** Ismaël Morin-Poulard, Manon Destalminil-Letourneau, Laetitia Bataillé, Jean-Louis Frendo, Gaëlle Lebreton, Nathalie Vanzo, Michèle Crozatier

**Affiliations:** ^1^ Unité de Biologie Moléculaire et Cellulaire et du Développement (MCD), Centre de Biologie Intégrative (CBI), Université de Toulouse UMR 5077/CNRS, Toulouse, France; ^2^ CNRS, INSERM, IGDR (Institut de Génétique et Développement de Rennes), UMR6290, ERL U1305, Rennes, France; ^3^ INSERM U1301, CNRS 5070, Université de Toulouse, Toulouse, France

**Keywords:** blood bi-potent progenitor, nephrocyte, lymph gland ontogeny, hematopoiesis, *Drosophila*, Klf15

## Abstract

The *Drosophila* lymph gland is the larval hematopoietic organ and is aligned along the anterior part of the cardiovascular system, composed of cardiac cells, that form the cardiac tube and its associated pericardial cells or nephrocytes. By the end of embryogenesis the lymph gland is composed of a single pair of lobes. Two additional pairs of posterior lobes develop during larval development to contribute to the mature lymph gland. In this study we describe the ontogeny of lymph gland posterior lobes during larval development and identify the genetic basis of the process. By lineage tracing we show here that each posterior lobe originates from three embryonic pericardial cells, thus establishing a bivalent blood cell/nephrocyte potential for a subset of embryonic pericardial cells. The posterior lobes of L3 larvae posterior lobes are composed of heterogeneous blood progenitors and their diversity is progressively built during larval development. We further establish that in larvae, homeotic genes and the transcription factor Klf15 regulate the choice between blood cell and nephrocyte fates. Our data underline the sequential production of blood cell progenitors during larval development.

## Introduction

**FIGURE 1:**
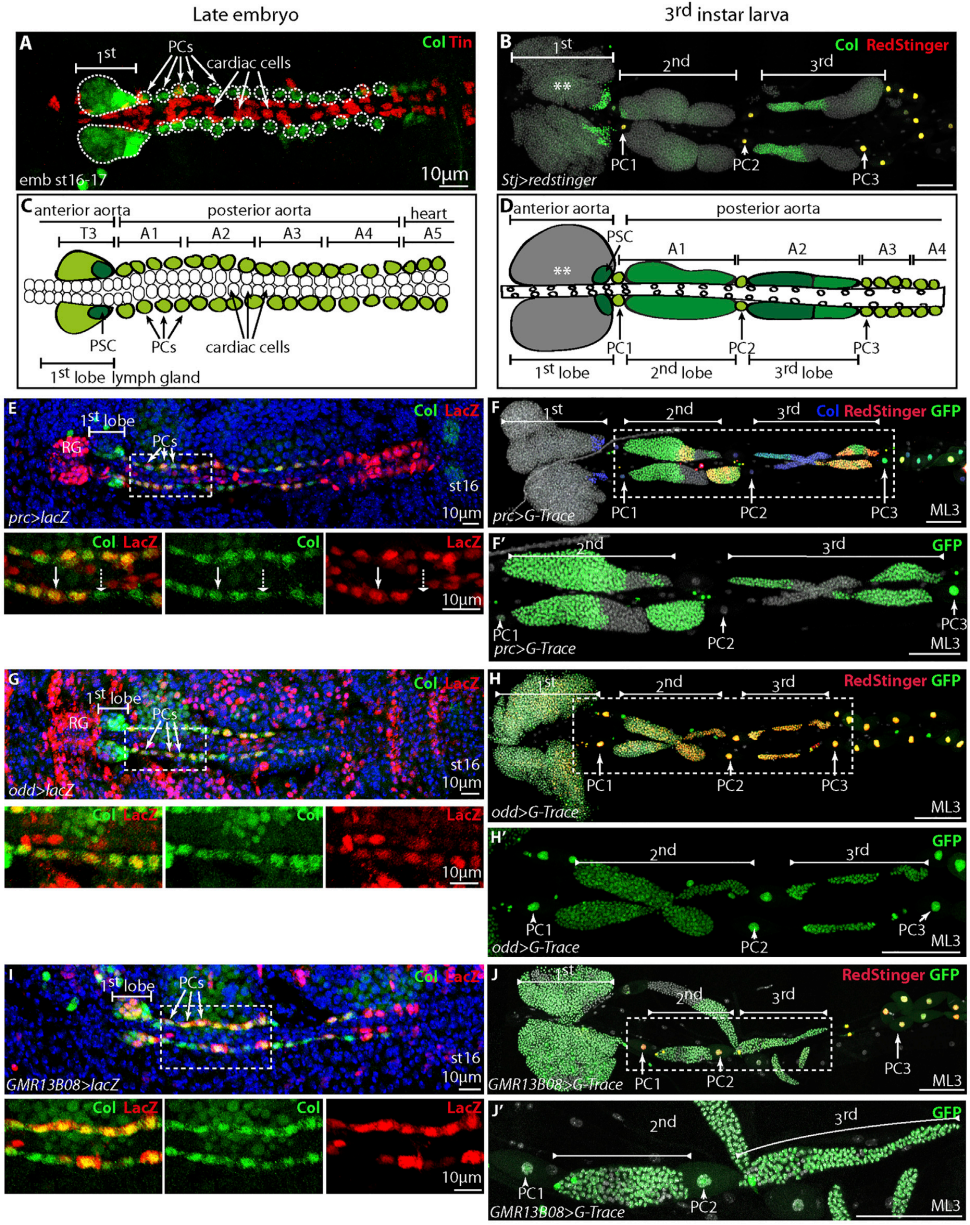
*Drosophila* embryonic and larval cardiovascular systems. Lymph gland posterior lobes originate from embryonic pericardial cells **(A,B)** Lymph gland in stage 16–17 embryo **(A)** and third instar larva **(B)**, Col expression is in green. **(A)** Col is mainly expressed in the PSC and in a subset of pericardial cells, cardiac cells express Tinman (Tin) (red). **(B)** Col is expressed in the PSC and progenitors (**) in anterior lobes, in subsets of blood cells in posterior lobes, and in all larval pericardial cells/nephrocytes which express RedStinger under the *Straight jacket-Gal4* (*Stj*) driver (red). **(C,D)** Schematic representation of the cardiovascular system in embryo **(C)** and L3 larvae **(D)**. Cardiac cells (white circles) and pericardial cells (PCs) expressing Col in green. Anterior (first) and posterior lobes (second and third) are indicated. Col expression in green, and dark green when Col expression levels are higher. **(E,G,I)** Cardiovascular system in stage 16 embryos, Col expression in green, LacZ (red) corresponds to β−galactosidase expressed under the *pericardin-Gal4* (*prc*) driver **(E)**, *odd-Gal4* **(G)** and *GMR 13B08-Gal4* (*col-Gal4*, I) drivers and Dapi (blue). White arrows indicate pericardial cells (PCs); the dashed arrow indicates PC with barely any GFP expression, and RG indicates the ring gland. **(F, F’, H, H’, J, J’)** Lymph glands of mid L3 larvae (ML3) and G-Trace experiments performed with *prc-Gal4* [*prc > G-Trace,* (F-F)’], *odd-Gal4* [*odd > G-Trace*, **(H,H’)**], and *GMR13B08-Gal4* for *Col* [*GMR13B08>G-Trace*, **(J, J’)**] drivers. **(F’, H’, J’)** are enlargements of **(F, H, J)**, respectively. **(F,H,J)** GFP (green) marks *prc* **(F, F’)**, *odd* **(H,H’)** and *col* **(J, J’)** cell lineage, while RedStinger (red) indicates ongoing expression of *prc* **(F)**, *odd* **(H)** and *col* **(J)** *Gal4* drivers at the moment of the experiment; in **(F)** Col is blue. **(E,G,I)** nuclei labelled with Dapi (blue) and scale bars = 100 µm, if not specified otherwise.

The *Drosophila* dorsal vessel is formed during embryogenesis and is required in larvae for the main heart function, i.e., ensuring hemolymph circulation throughout the organism (Rugendorf et al., 1994). The dorsal vessel is composed of two major cell types: the cardiomyocytes that form the cardiac tube, and associated non-contractile pericardial cells (PCs), also called nephrocytes (Zaffran and Frasch, 2002; Mandal et al., 2004). The *Drosophila* nephrocyte has two functions, flitration and protein re-absorption, which are similar to those of the podocyte, a highly specialized cell-type crucial for glomerular filtration in mammalian kidneys (Weavers et al., 2009; Zhuang et al., 2009; Zhang et al., 2013; Tutor et al., 2014). The *Drosophila* dorsal vessel derives from progenitors that are specified in the embryonic lateral mesoderm. By the end of embryogenesis, the cardiac tube is divided along the anterior/posterior (A/P) axis into the aorta (anterior part) and the heart proper (abdominal segments A5–A7). The aorta is further divided into two parts: the anterior aorta (from the anterior end to the T3 thoracic segment), and the posterior aorta (A1-A4 abdominal segments) (Perrin et al., 2004) (Figures 1A,C). Approximately 110–130 pericardial cells along the abdominal segments flank each side of the embryonic heart (Monier et al., 2007). They can be subdivided into different subtypes based on their expression of different transcription factors, most of which likely undergo histolysis at the larval stage, with only around 38–40 PCs persisting in third instar larvae, and which are called nephrocytes (Rizki and Rizki, 1978; Sellin et al., 2006; Monier et al., 2007; Bataille et al., 2020).

The lymph gland, which is the larval hematopoietic organ, is associated to the anterior aorta (Perrin et al., 2004; Monier et al., 2007) (Figures 1A,C). In third instar larvae (L3) the lymph gland consists of one pair of anterior lobes (first lobe) localized in T2 and T3 thoracic segments, and two pairs of smaller posterior lobes which extend into abdominal segments A1-A2 (Crozatier et al., 2004) (Figures 1B,D). Lymph gland anterior and posterior lobes are separated from each other by one pericardial cell, which displays abundant cytoplasm and a large nucleus resulting from polytenization (Sellin et al., 2006). The first lobes are composed of a heterogeneous population of hematopoietic progenitors and differentiated blood cells, also called hemocytes (Jung et al., 2005; Cho et al., 2020; Morin-Poulard et al., 2021), and the balance between progenitors and differentiated blood cells (homeostasis) is controlled by the Posterior Signaling Center (PSC) and the cardiac tube, which play roles reminiscent of the endosteal and perivascular niches in mammals (Krzemien et al., 2010; Letourneau et al., 2016; Banerjee et al., 2019; Destalminil-Letourneau et al., 2021; Morin-Poulard et al., 2021). Unlike the first lobe, posterior lobes are only composed of hematopoietic progenitors (Jung et al., 2005; Kanwal et al., 2021; Rodrigues et al., 2021).

The two pairs of posterior lobes, hereafter referred to as second and third lobes, start to develop at the end of the first larval instar (L1), beginning of the second larval instar (L2) (Sellin et al., 2006). A recent study (Rodrigues et al., 2021) shows that posterior lobes are composed of a heterogeneous pool of progenitors, which in response to wasp parasitism activates STAT92E and remain undifferentiated. In an independent study, Kanwal et al. propose that a subset of cells localized in the third lobes and strongly expressing Ubx and Col, is required to maintain blood progenitor state in posterior lobes (Kanwal et al., 2021). In this study we investigated posterior lobe ontogeny and cell content, from L1 to L3. By lineage tracing we first establish that posterior lobe blood cells originate from embryonic bi-potent blood/pericardial cells. We then show that while blood/pericardial bi-potent cells express the same markers at the L1 stage, later modifications in these expressions result in L3 larvae posterior lobes which are composed of heterogeneous blood cell progenitors, and this diversity is progressively set up during larval development. Finally, we investigated the molecular basis for blood cell/nephrocyte cell fate decision. Our data highlight how, at the larval stage, homeotic genes and the transcription factor Klf15 play an important role in the cellular choice between either nephrocyte or blood cell fates.

## Materials and Methods

### Fly Strains

*w* ^*1118*^ (wild type, *WT*), *stj-Gal4* (GMR 76H09, BDSC 46974), *prc-Gal4* (Chartier et al., 2002), o*dd-Gal4* (Levy and Larsen, 2013), *GRM13B08-Gal4* (BDSC 48546), *UAS-Nls-LacZ* (BDSC 6451), *UAS-G-Trace* (BDSC 28280), *Hs-Flpm5* (BDSC 81068), *UAS-Flybow1.1b* (BDSC 56803), *UAS-mCD8-GFP* (BDSC5137), *Handc-mCherry* (Paululat and Heinisch, 2012), *Handc-GFP* (Sellin et al., 2006), *76E11-Gal4* (BDSC 39933), *UAS-Antp* (BDSC 7301), *UAS-Ubx* and *UAS-AbdA* (Michelson, 1994), *UAS-RNAi-Ubx* (BDSC 34993), *Klf15* mutant (BDSC 18979), *UAS-RNAi-Klf15* (BDSC 27075), *UAS-FLP* (BDSC 8205), *Act-FRT-Stop-FRT-Gal4 UAS-GFP* (BDSC 4411), *eme-Gal4* (Han et al., 2002).

### Antibodies and Immunostaining

Embryos were fixed as described in (Crozatier et al., 1996). Lymph glands were dissected and processed as described in (Krzemien et al., 2007). Larva (Louradour et al., 2017) and embryo (Crozatier et al., 1996) immunostainings were performed as previously described. Antibodies used were mouse anti-Col (1/100) (Krzemien et al., 2007), rabbit-anti-Tin (1/100, Manfred Frasch, Erlangen, Germany), chicken anti-βgal (1/1000, Abcam), rabbit-anti-H3P (1/200, Cell Signalling Technology). Secondary antibodies were Alexa Fluor-488, -555 and -647 conjugated antibodies (1/1000, Molecular Probes) and goat anti-Chicken Alexa Fluor-488 (1/800, Molecular Probes). Nuclei were labeled with DAPI (1 μg/ml, Sigma).

### Confocal Imaging

Pictures were taken with a SP8 confocal (Leica) with a ×40 objective, or a ×63 objective for L1 lymph glands. 1µm or optimized z-size steps were used for imaging and 3D reconstruction for quantification, respectively. Multiple positions were used and stitching was performed using the Grid/Collection stitching Fiji plugin (Preibisch et al., 2009).

### Lineage Tracing Experiments

*Gal4* driver flies were crossed with *G-Trace* or *Act-FRT-Stop-FRT-Gal4 UAS-GFP* flies. Crosses and subsequent raising of larvae until late embryo/early L1 stage were performed at 22°C, before shifting larvae to 25°C until their dissection at the L3 stage.

### Multicolor Clone Production

*Hs-Flpm5; odd > Flybow1.1b* flies laid eggs 6 h at 25°C. Crosses and subsequent raising of larvae were performed at 25°C. At late embryo/early L1 stage (18 h after end of egg laying) a 2 h heat shock at 38.5°C was performed. Larvae were grown at 25°C during 72 h before their dissection. At least 15 lymph glands per genotype were analyzed, and experiments were reproduced at least three times. Among 45 lymph glands analyzed, seven independent clones expressing either mCherry or mCitrine were generated.

### RNAi Treatments

Controls correspond to Gal4 drivers crossed with *w* ^*1118*^. In all experiments, crosses and subsequent raising of larvae until late embryo/early L1 stage were performed at 22°C, before shifting larvae to 29°C until their dissection at the L3 stage. At least eight lymph glands were analyzed, and experiments were reproduced three times.

### Quantification of Col, prc > GFP and Handc-mCherry

Each posterior lobe was divided into three domains of equivalent size. For nuclear staining, Dapi labelling was used to define ROIs (regions of interest) in posterior lobes using Fiji software. The nuclear mean intensity of Col, prc > GFP and Handc-mCherry in ROIs was quantified using Fiji software. For prc > GFP staining in L1, the GFP mean intensity was measured for the whole cell. At least 6, 10 and 10 lymph glands for L1, ML2, and ML3 larvae were analyzed, respectively, and experiments were reproduced twice. Statistical analysis: one way ANOVA test (Dunnett test) performed using GraphPad Prism 5 software and PC1 was used as the reference.

### Mitotic Index Measurement

For counting mitotic cells, anti-H3P staining was performed. Since lymph gland size fluctuates from one larva to another, even in synchronized larvae, measuring the mitotic index is therefore the most reliable way to quantify proliferation. The mitotic index in posterior lobes was measured by dividing the number of H3P^+^ cells by the total number of posterior lobe cells. Each posterior lobe was cropped manually, and both H3P^+^ cells and nuclei, labelled by DAPI, were quantified using the 3DimageSuite Fiji plugin (Ollion et al., 2013). At least 12 lymph glands were analyzed, and experiments were reproduced twice.

## Results

### Specific Embryonic Pericardial Cells Give Rise to Blood Cell Progenitors at the Larval Stage

The number of pericardial cells drops dramatically during larval development, in part due to cell death in L1 larvae (Sellin et al., 2006). In order to assess whether embryonic pericardial cells contribute to posterior lobes formation, we performed lineage tracing experiments using the G-TRACE system (Evans et al., 2009) and embryonic pericardial cell Gal4 drivers. In this system, the RedStinger protein reveals the Gal4 driver real-time expression, while the nEGFP (nuclear GFP) labels the progeny. In late embryo, *pericardin (prc)*, which encodes a type IV collagen-like extracellular matrix protein, is expressed in pericardial cells and not in anterior lymph gland lobes (Perrin et al., 2004). *pericardin-Gal4* driver (*prc-Gal4*) reproduces *pericardin* expression (Chartier et al., 2002) (Figure 1E). In G-TRACE lineage experiments with *prc-Gal4,* RedStinger was barely detected in mid-L3 pericardial cells, but a strong staining was observed in groups of posterior lobe cells, indicating that at mid-L3 stage *prc–Gal4* is expressed in a subset of posterior lobe cells (Figure 1F). nEGFP was detected in pericardial cells as well as in most posterior lobe cells (Figures 1F,F’ and Supplementary Figures S1A,A’), suggesting that embryonic pericardial cells give rise to posterior lobe hemocytes. Not all posterior lobe cells expressed nEGFP, probably because this driver is not expressed at high levels in all embryonic pericardial cells (Figure 1E). Similar conclusions were obtained by performing lineage tracing with the Flp-out method (UAS Flp; Actin5C-FRT-stop-FRT-Gal4, UAS-mCD8GFP) and the *prc-Gal4* driver (Supplementary Figures S1B,B’). G-TRACE lineage experiments were performed with *76E11-Gal4*, another Gal4 driver expressed in embryonic pericardial cells, as well as first lobe and cardiac cells (Supplementary Figure S1C). All larval pericardial and posterior lobe cells expressed nEGFP, further supporting that posterior lobe cells are generated from embryonic pericardial cells (Supplementary Figures S1E,E’).

### Embryonic Odd^+^Col^+^ Pericardial Cells Have a Blood Cell/Nephrocyte Bipotential

Ten pericardial cells are found per embryonic abdominal hemi-segment, and they that can be divided into four different subtypes, based on their expression of different combinations of transcription factors including Tinman (Tin), Even-skipped (Eve), Odd-skipped (Odd), Seven-up (Svp) and Ladybird (Lb) (Monier et al., 2007; Das et al., 2008; Ocorr et al., 2014; Ahmad, 2017) (Supplementary Figure S1D). This raised the question of which subtypes of embryonic pericardial cells give rise to larval posterior lymph gland cells. In stage 16 embryo, four out of ten pericardial cells per hemi-segment express Odd (Ward and Coulter, 2000; Ahmad, 2017) (Supplementary Figure S1D). Collier/Knot (Col/Kn) was reported to be expressed, together with Odd, during formation of the embryonic lymph gland (Crozatier et al., 2004), and Col is expressed in some embryonic pericardial cells (Figures 1A,C). We therefore asked whether Odd-positive cells are the ones expressing Col. Using the *odd-Gal4* driver as a proxy of Odd expression (Levy and Larsen, 2013), we found that all embryonic pericardial cells expressing LacZ, under the control of *odd-Gal4,* express Col (Figure 1G). We then asked whether Odd^+^Col^+^ embryonic pericardial cells contribute to posterior lobe formation. To identify the progeny of these Odd^+^Col^+^ embryonic pericardial cells, we performed lineage tracing experiments using the *odd-Gal4* and the GMR13B08 *col-Gal4* drivers, the latter reproducing Col expression in embryonic pericardial cells (Carayon et al., 2020) (Figure 1I). Lineage tracing with Odd and Col drivers indicates that nEGFP is expressed in posterior lobe cells, as well as in all larval pericardial cells (Figures 1H,H’,J,J’). This data establishes that Odd^+^Col^+^ embryonic pericardial cells give rise to posterior lobe blood cells in A1-A2 segments, as well as to all larval nephrocytes. This also suggests that most embryonic pericardial cells die in the course of larval development. To verify this point, we performed G-TRACE lineage tracing, using the *eme-*Gal4 driver for Eve embryonic pericardial cells (Han et al., 2002). We never detected nephrocytes expressing nEGFP in L3 larvae with this driver, indicating that Eve pericardial cells do not survive larval development (Supplementary Figures S1F,F’). In this analysis, we did not look at the few Eve expressing pericardial cells found in thoracic segments, which give rise in adults to wing hearts in the thorax (Togel et al., 2008). In conclusion, Odd^+^Col^+^ embryonic pericardial cells survive in larvae and give rise to both posterior lobe lymph gland cells and larval nephrocytes.

### Each Posterior Lobe Originates From Three Embryonic Pericardial Cells

**FIGURE 2:**
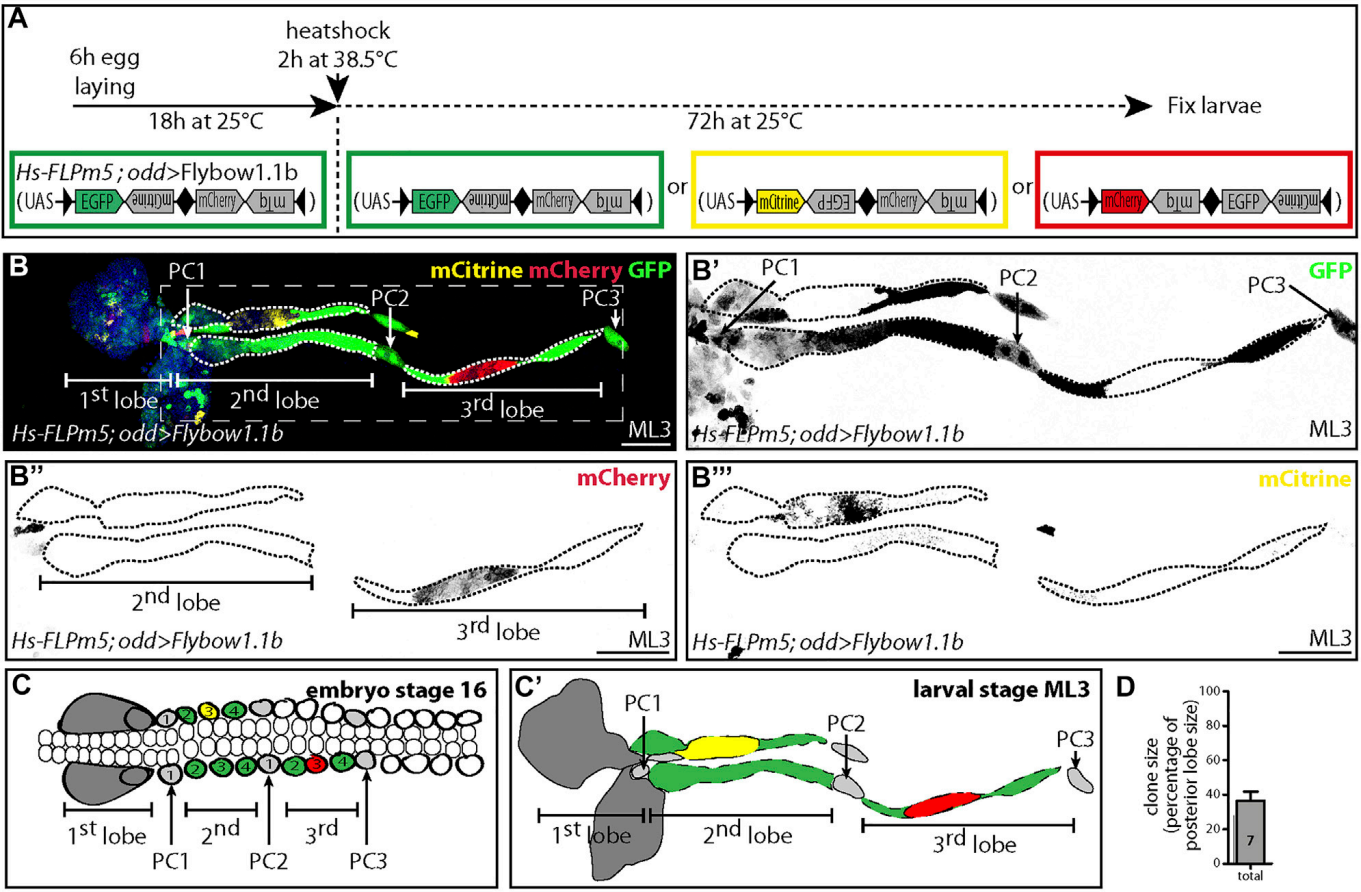
Each posterior lobe is formed from three embryonic pericardial cells **(A)** Schematic of flybow variants. Pairs of fluorescent protein-encoding cDNAs arranged in opposing orientations and flanked by FRT sites (black arrowheads). Constructs cloned into a UAS vector containing ten UAS sites and called Flybow1.1b. FLPm5 under the control of the heat-shock promoter (Hs-FLPm5) induces recombination at FRT sites. Three fluorescent proteins can be expressed: EGFP (green), mCitrine (yellow) and mCherry (red). **(B–B”’)** In this vector mTq (turquoise) cannot be visualized (Hadjieconomou et al., 2011)**.** Confocal images of midL3 (ML3) larval lymph gland expressing different fluorescent proteins. **(B)** three colors are shown, **(B’)** GFP (black), **(B”)** mCherry (black), **(B’”)** mcitrine (black). Nuclei labelled with Dapi [blue in **(B)**]. **(C,C’)** Schematic representation of lymph gland first lobes, cardiac and pericardial cells in stage 16 embryo **(C)** and ML3 lymph gland **(C’)**. In the second lobe, the cell cluster expressing mcitrine (yellow) derives from one embryonic pericardial cell [number 3 and in yellow in**(C)**]. The two other embryonic pericardial cells contributing to this second lobe both express GFP [green and labelled 2 and 4 in **(C)**]. In the third lobe, the cell cluster expressing mCherry (red) corresponds to progeny of one embryonic pericardial cell [red and labelled 3 in **(C)**]. The two other embryonic pericardial cells contributing to this 3rd lobe both express GFP [green and are labelled 2 and 4 in **(C)**]. **(D)** Clone size is given as a percentage relative to total posterior lobe size. Only mCitrine and mCherry clones are given. A total of seven clones were analyzed corresponding to two, four and one clones localized in anterior, middle and posterior part of posterior lobe.

To further study the formation of posterior lobes during larval development, we performed clonal analysis by adapting the multicolor lineage tracing technic called “rainbow” or “confetti” (Livet et al., 2007), called “Flybow” in *Drosophila*. This technic combines the binary Gal4/UAS system to regulate membrane-tethered fluorescent protein expression and a modified heat shock inducible Flipase (*Hs-FLPm5*) that catalyzes recombination at the Flipase Recognition Target (FRT) sites flanking each fluorescent cassette (Hadjieconomou et al., 2011; Shimosako et al., 2014). Recombination at FRT sites leads to stochastic expression of different fluorescent proteins in a clonal manner, enabling cell lineage tracing and in turn determination of the parental origin of cellular progeny. We used the Flybow cassette containing four genes in tandem, coding different fluorescent proteins and the *odd-Gal4* driver. EGFP is the default fluorescent protein, whereas recombination events lead to mCitrine and mCherry expression (Hadjieconomou et al., 2011) (Figure 2A). Heat shock was delivered at the end of embryogenesis to favor labelling of the complete pericardial cell lineage, and lymph glands were analyzed at the L3 instar. One example of lymph gland staining is shown (Figure 2B). EGFP is expressed in a large subset of cells, as it reflects the expression of the Gal4 driver (Figures 2B,B’). One mCherry clone can be observed in one third posterior lobe (Figure 2B”), while an independent clone expressing mCitrine is observed in one second posterior lobe (Figure 2B”’). The third posterior lobe is composed of three clones of similar size, one clone in the middle of the lobe expressing mCherry and surrounded by the two other clones expressing EGFP (Figure 2B”), suggesting that three embryonic pericardial cells contribute to the formation of the third posterior lobe. For the second posterior lobe similar results are observed (Figures 2B’,B”’), establishing that three embryonic pericardial cells give rise to the second posterior lobe. Quantifications of the size of all clones obtained are performed. On average the size of each clone corresponds to one third of the total lobe size (Figure 2D). Altogether, these data establish that in A1-A2 segments, four pericardial cells per hemisegment give rise to one larval nephrocyte, while the three others contribute to the formation of one posterior lobe. In addition, inside each posterior lobe, each embryonic pericardial cell proliferates at a similar rate during larval development and thus contributes equally to posterior lobe formation (Figures 2C,C’).

### Inter and Intra Posterior Lobe Cell Heterogeneity is Established During Larval Development

**FIGURE 3:**
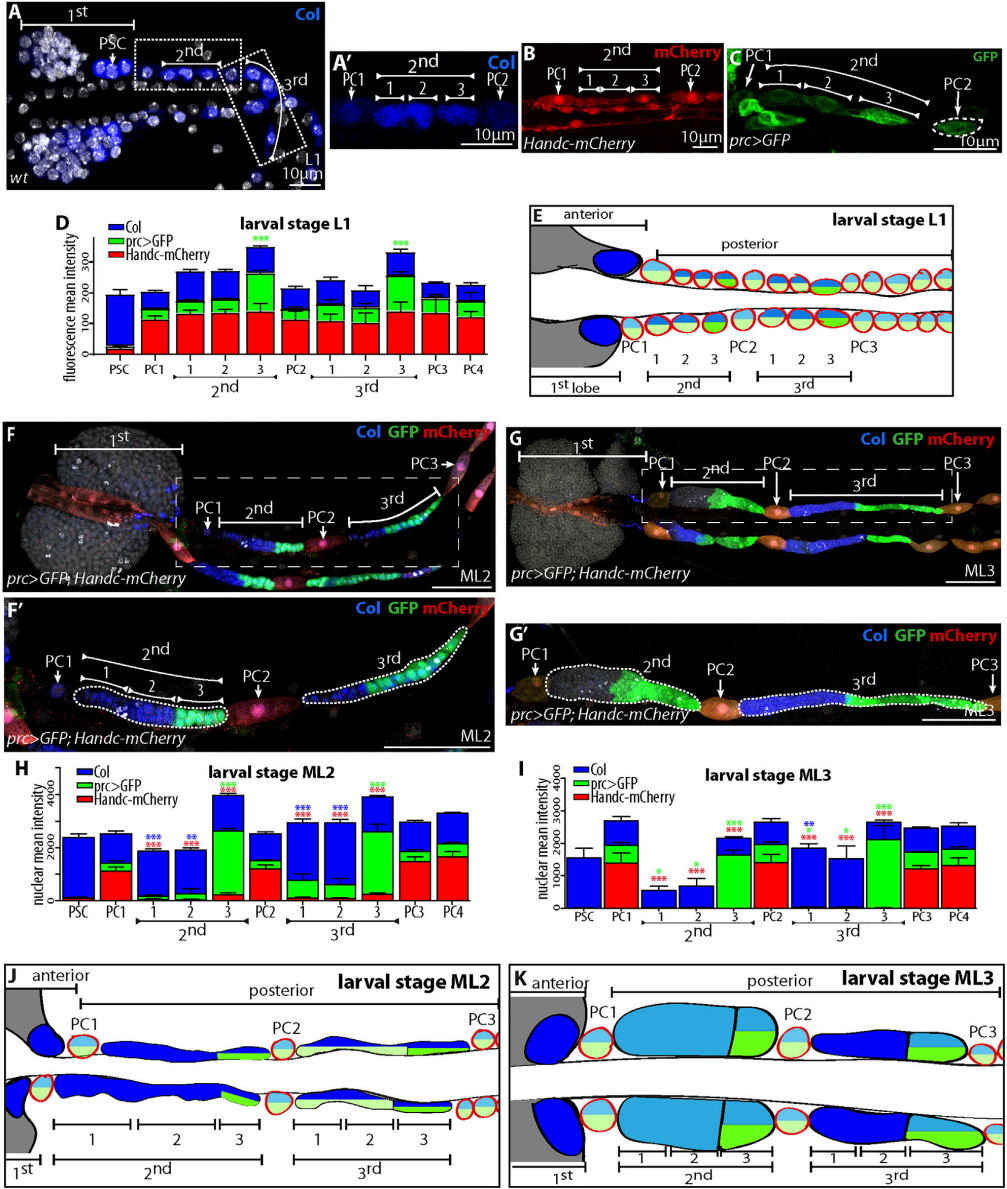
Heterogeneity in posterior lobe cells established during larval development **(A–C)** Lymph gland of first instar larvae (L1). **(A,A’)** Col (blue) expressed in the PSC and in progenitors of the first lobe, as well as in all PCs, Dapi (white) labels nuclei. **(A’)** Enlarged views where PC1 and PC2 are indicated, as well as the three bipotent nephrocyte/blood cells giving rise to the second lobe. Col (blue) is expressed in both PCs and bipotent nephrocyte/blood cells. **(B)** Handc-mCherry (red) labels cardiac and pericardial cells. **(C)** *prc > GFP* (green) labels pericardial cells. GFP is expressed at higher levels in third bi-potent nephrocyte/blood cells. **(D)** Quantification of Col, *prc > GFP* and Handc-mCherry. **(E)** Schematic representation of lymph gland posterior lobes in L1 larvae. Col is blue, or dark blue for higher expression levels, *prc > GFP* is green, and Handc-mCherry is represented by red circles. **(F,F’,G,G’)** *prc > GFP* (green) lymph gland of mid second instar larvae [ML2, **(F,F’)**] and mid third instar larvae L3 [ML3, **(G,G’)**], *Handc-mCherry* (red) labels cardiac and pericardial cells and Col is in blue. **(F’, G’)** enlarged views, GFP (*prc > GFP*, green), Handc-mCherry (red) and Col (blue). **(H,I)** Each posterior lobe is divided into three subgroups of similar size for quantification. Quantification of Col, *prc > GFP* and Handc-mCherry. For all quantifications and figures, statistical analysis one way ANOVA test (Dunett test, PC1 as a reference) performed using GraphPad Prism 5 software. Error bars represents SEM and *p < 0,1, **p < 0,01, ***p < 0.001; when no significant difference: not shown. **(J,K)** Schematic representation of larval lymph gland posterior lobes in ML2 **(J)** and ML3 **(K)** larvae. Pericardial cells circled in red; Col in blue, or dark blue for higher expression levels, *prc > GFP* is green or dark green for higher expression levels.

In L3 larvae, posterior lobes are composed of hematopoietic progenitors (Crozatier et al., 2004; Jung et al., 2005; Grigorian et al., 2011; Benmimoun et al., 2015; Oyallon et al., 2016; Kanwal et al., 2021; Rodrigues et al., 2021). At this stage, and in agreement with previous reports (Kanwal et al., 2021; Rodrigues et al., 2021), Col is expressed in a subset of posterior lobe cells and its expression pattern is complex, since some progenitors express it at high levels, whereas it is barely detected in others (Figures 1B,D). In L1 larvae Col is expressed both in future larval nephrocytes and posterior lobe progenitors (Figures 3A,A’). This observation indicates that a diversification in posterior lobe progenitors could occur during larval development. To further analyze this, we followed throughout larval development, the expression patterns of three markers of embryonic pericardial cells, Col, prc > GFP and Handc-mCherry (Figures 3A–C). In L1 larvae, Col is expressed at higher levels in hematopoietic precursors compared to nephrocytes (Figures 3A,A’,D). Handc-mCherry is expressed at similar levels both in nephrocytes and hematopoietic precursors (Figure 3D), while *prc > GFP* is expressed at higher levels in the posterior region of posterior lobes (Figures 3C,D). At mid L2 (ML2) and mid L3 (ML3) stages (Figures 3F,G’), larval nephrocytes can be identified based on their morphology, and the expression of the three markers in these cells is not modified compared to the L1 stage (Figures 3H,I). To follow the diversification of cell expression programs in posterior lobes, we subdivided each lobe into three sub-domains of equivalent size (see Figure 3F’’) and measured for each subdomain the expression of the three markers. At mid L2 and mid L3 stages, in the second lobe, a decrease in Col and Handc-mCherry levels is observed in all three sub-domains (Figures 3H,I). For *prc > GFP*, its expression decreases in subdomains one and two but remains high in sub-domain 3 (Figures 3H,I). These data reveal intra lobe blood cell heterogeneity. For the third lobe, *prc > GFP* and Handc-mCherry expression vary in the same way as observed for the second lobe. In contrast, a clear difference for Col expression is observed between the second and third lobes. In the third lobe, Col expression is maintained at high levels in sub-domains 1 and 2, while it decreases in sub-domain 3 (Figures 3G–I), revealing heterogeneity in between the second and third lobes (Figures 3J,K). In conclusion, posterior lobe progenitors are heterogeneous, with their diversity being gradually implanted during larval development.

### Heterochrony in Posterior Lobe Growth During Larval Development

**FIGURE 4:**
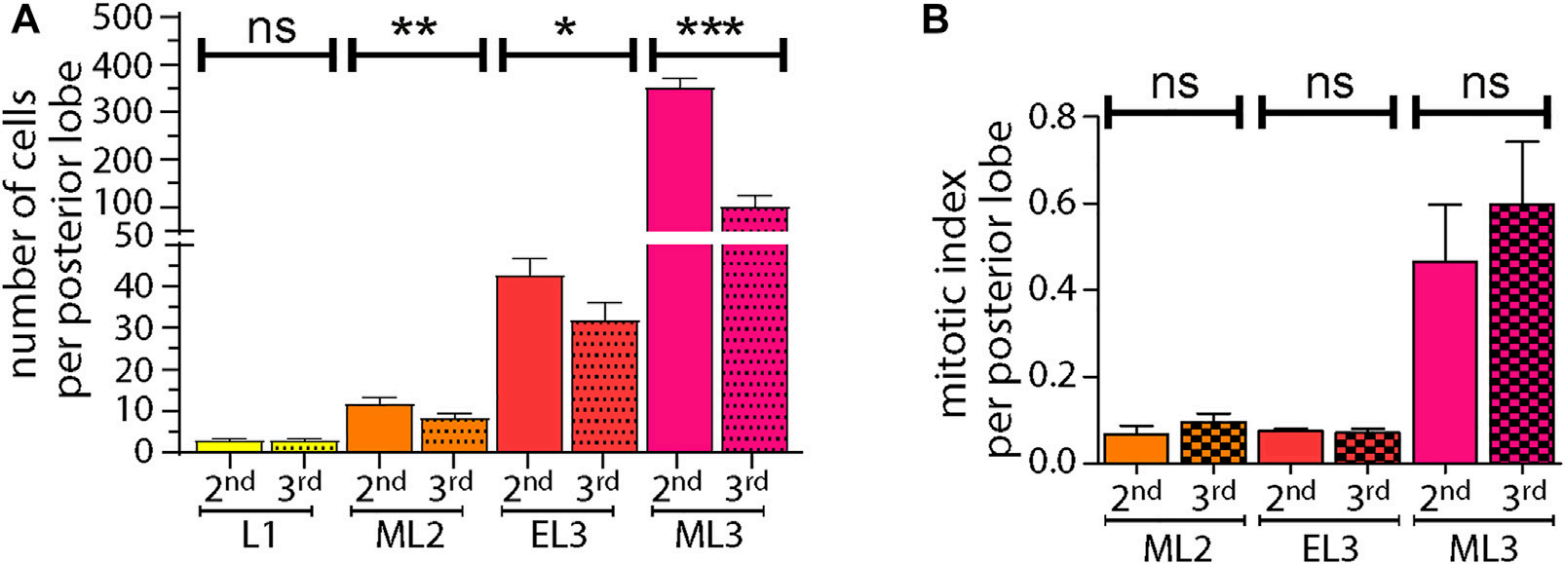
Posterior lobe growth during larval development. **(A)** Quantifications of posterior lobe cell numbers during larval development. *p < 0,1; **p < 0.001,***p < 0.001 and ns (not significant). **(B)** Quantifications of mitotic index in posterior lobes during larval development.

We wondered whether the two pairs of posterior lobes were growing at a similar rate during larval development, and therefore counted the number of posterior lobe cells during larval development (Figure 4A). In L1 larvae, three embryonic pericardial cells contribute to the formation of one posterior lobe. In mid-L2 larvae, an average of 12 cells constitute the second lobe, while the third lobe is composed of only nine cells. These data indicate that proliferation in the L2 stage starts somewhat earlier in the second lobe compared to the third lobe. The difference in cell numbers increases significantly between the second and third lobes in early L3. In mid-L3 larvae, about 350 cells compose the second lobe, while only 100 cells compose the third lobe. By calculating the mitotic index for each posterior lobe in the L3 larval stage, we found no significant difference, suggesting that there might be a transient burst of proliferation in the second lobe, resulting in their different cell number, and not detectable in a snapshot of H3P^+^ cells. Nevertheless, in mid-L3 larvae, cell division increases in both lobes (Figure 4B), which is in agreement with published data (Rodrigues et al., 2021).

### *Hox* Genes and *Klf15* Contribute to Carry out Nephrocyte or Blood Fate at the Larval Stage

**FIGURE 5:**
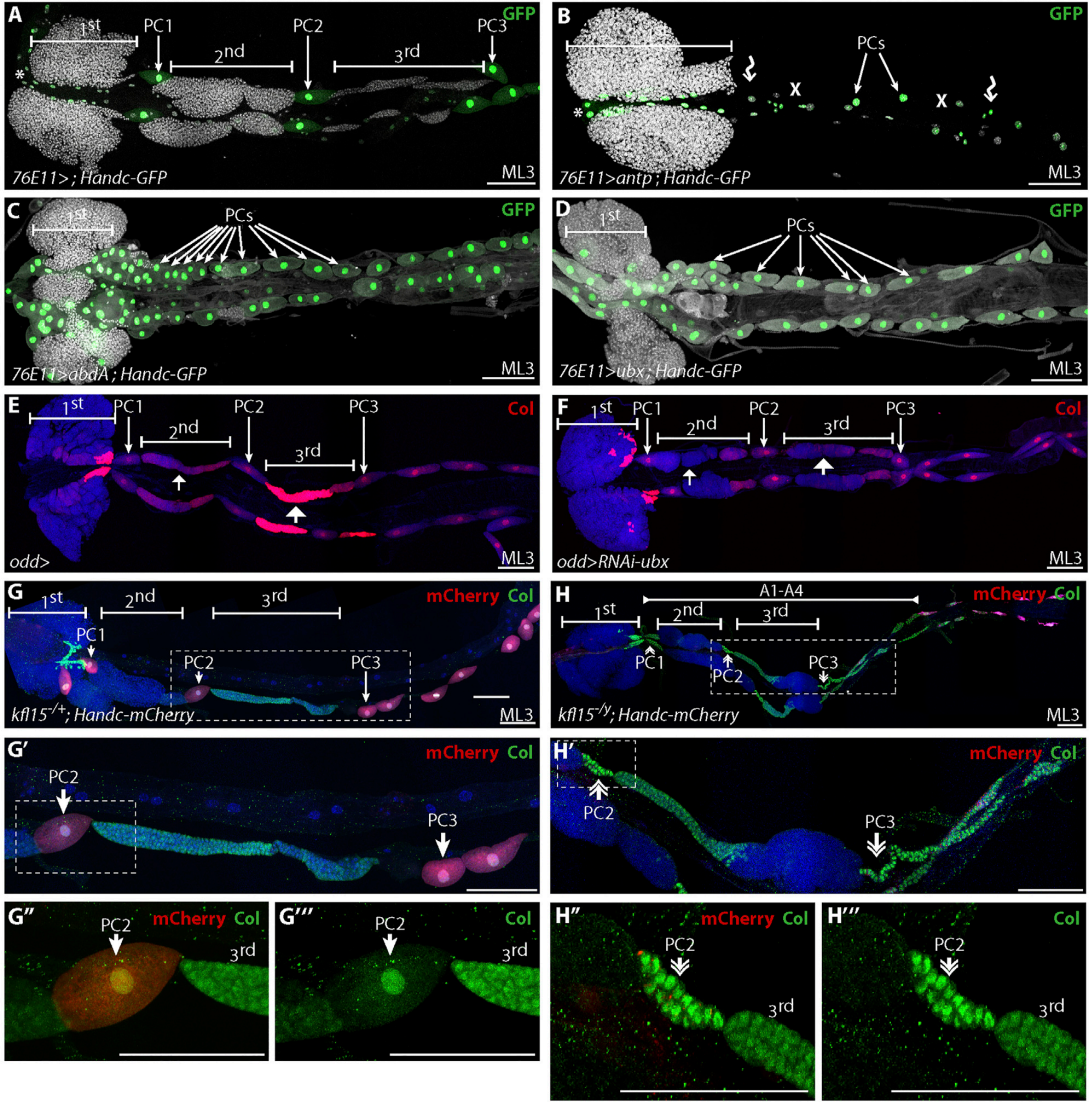
Ubx and Klf15 required at larval stage to regulate blood cell and nephrocyte fate **(A–D)** Mid L3 larvae (ML3) lymph glands expressing Handc-GFP (green), which labels cardiac (*) and pericardial cells (white arrow), with the *76E11-Gal4* driver, and Dapi (white) labels nuclei. **(B)** *antp* overexpression leads to loss of both posterior lobes (wavy arrow) and most pericardial cells (X). **(C)** *abdA* or **(D)** *ubx* overexpression leads to loss of posterior lobes, together with formation of supernumerary larval pericardial cells (PCs). **(E,F)** Col (red) immunostaining in the control **(E)** and when *ubx* is knocked down in pericardial cells, with the *odd-Gal4* driver **(F)**. No difference in pericardial cells and posterior lobe formation is observed compared to the control. Col expression (small and large white arrows) has decreased in posterior lobes compared to the control. **(G,H”’)** Mid L3 lymph gland of control *Klf15/+* **(G-G”’)** and *Klf15* mutant [*Klf15/Y,* **(H-H”’)**]. Col expression in green, Handc-mcherry which labels larval pericardial cells in red, and Dapi in blue labels nuclei. **(G’,H’)** are enlargements of **G and H**, respectively. **(G”,G”’,H”,H”’)** are enlargements of **(G’,H’)**, respectively. *Klf15* mutant **(H-H”’)** pericardial cells are abnormal and resemble novel posterior lobes, while second and third lobes resemble the control **(G-G”’)**. In the posterior aorta (A1-A4) of *Klf15* mutant larvae, pericardial cells are transformed into blood cells (double arrowhead), expressing high levels of Col without expressing Handc-mCherry, a marker for pericardial cells.

Expression of the homeotic genes *Antennapedia* (*Antp*), *Ultrabithorax* (*Ubx*), *abdominal-A* (*abd-A*) and *Abdominal-B* (*Abd-B*) in distinct domains along the anterior/posterior axis (A/P), provides positional information that subdivides the dorsal vessel and associated tissues, including the lymph gland for review see (Lo and Frasch, 2003; Bataille et al., 2015). Ubx is expressed in A1-A4 segments, while Antp is expressed in T3, where the primary lobe of the embryonic lymph gland is localized. In embryos mutant for either *ubx* or both *ubx* and *abd-A*, the lymph gland is not restricted to thoracic segments but is expanded posteriorly into abdominal segments, indicating that during embryogenesis ubx/abd-A inhibits lymph gland formation in abdominal segments (Mandal et al., 2004; Perrin et al., 2004). Upon ectopic expression in the mesoderm of either *antp, ubx* or *abd-A*, anterior aorta is converted into posterior aorta, where lymph gland cells are missing and only pericardial cells are present (Perrin et al., 2004). We therefore wondered whether *antp, ubx* or *abd-A* could affect the choice between blood or nephrocyte fate at the larval stage. We ectopically expressed *antp, abd-A* or *ubx* in embryonic pericardial cells, once specified, using the *76E11-Gal4* driver. Upon *antp* ectopic expression, posterior lobes failed to form and most pericardial cells were lost (Figures 5A,B), suggesting that pericardial cells died and as a consequence posterior lobes failed to form. Upon ectopic expression of *abd-A* or *ubx,* no posterior lobes formed, and additional pericardial cells were observed compared to the control (Figures 5A,C,D). Excess pericardial cells were where posterior lobes should have formed, suggesting that embryonic pericardial cells gave rise to larval nephrocytes at the expense of blood cells. Taken together, these results suggest that the over or ectopic expression of either *ubx* or *abd-A* can prevent embryonic pericardial cells from choosing a blood cell fate at the larval stage. Knocking-down *ubx* in pericardial cells, once they had been specified in embryo, had no impact on either nephrocyte or posterior lobe formation (Figure 5F). However, high Col expression in a subset of cells in the third lobe was lost (compare Figures 5E,F), confirming published data indicating that *ubx* regulates Col expression in those cells (Kanwal et al., 2021). These data further establish a dual role for *ubx* in controlling blood fate during development. In embryo, *ubx* prevents blood cell fate in abdominal segments, and in L1-L2 larval stages *ubx* function has to be inhibited to allow posterior lobe formation from bi-potent blood/nephrocyte progenitors. *ubx is* expressed in a subset of posterior lobe cells in L3 larvae and at this developmental stage is required to maintain posterior lobe progenitors (Kanwal et al., 2021; Rodrigues et al., 2021).

What implements the choice between nephrocyte and blood cell fates at the larval stage? One candidate is the *Drosophila* orthologue of the mammalian, kidney Krüppel-like factor (Klf15), previously shown to be required for larval nephrocyte formation (Ivy et al., 2015). We investigated whether posterior lobe formation was affected in *Klf15* loss-of-function mutants. We found that Handc-mcherry was barely expressed in *Klf15* loss-of-function mutants in A1-A4 segments (Figures 5G,H). Some faint staining was detected outside A1-A4, in the most posterior larval nephrocytes, identified by their large size and big nuclei (Figure 5H). This data confirmed that larval nephrocytes are lost in *Klf15* mutants (Ivy et al., 2015). However, the most striking observation was along the posterior aorta (A1-A4 segments) and in place of larval nephrocytes, the formation of small cell clusters resembling posterior lobes (Figures 5H-H”). These cells are tightly packed together, they have a nuclear and cellular size similar to posterior lobe cells, and they express Col at high levels. Similar phenotypes are obtained when *Klf15* is knocked down by expressing *Klf15-*RNAi in pericardial cells with the *odd-Gal4* driver (Supplementary Figures S2B,B’). Taken together, these results suggest that in the posterior aorta of a *Klf15* mutant, ectopic posterior lobes form at the expense of larval pericardial cells. This reveals that all A1-A4 embryonic pericardial cells have the potential to become either nephrocyte or blood cells, and that the nephrocyte fate is dependent upon *Klf15* activity. Is *Klf15* expression sufficient to prevent blood cell fate and push embryonic pericardial cells to becoming nephrocytes? To address this point, we ectopically expressed *Klf15* in embryonic pericardial cells, using the *odd-Gal4* driver. No difference compared to the control was observed (Supplementary Figures S2A,C,C’), indicating that *Klf15* is required but not sufficient to promote nephrocyte fate in all embryonic pericardial cells.

## Discussion

**FIGURE 6:**
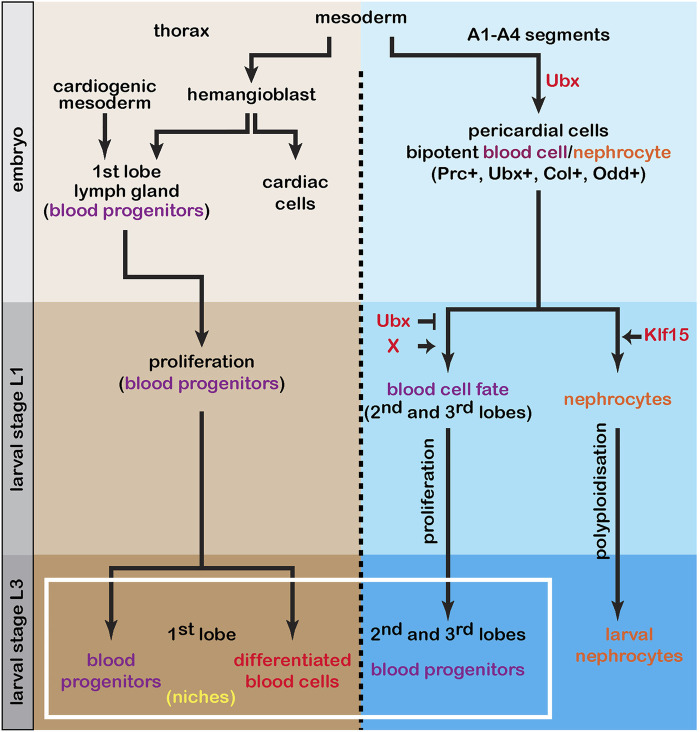
The lymph gland is a composite organ The first lymph gland lobe originates in the embryo from the cardio-mesoderm and hemangioblast. At the end of embryogenesis the lymph gland is composed of blood progenitors that will proliferate during larval stage. In third instar larvae, the first lobe is composed of both progenitors and differentiated blood cells. The balance between these two cell populations (homeostasis) is controlled by two niches. During embryogenesis, in A1-A4 segments and depending on Ubx, bi-potent blood cell/nephrocyte progenitors are specified. At larval stage L1, while larval nephrocytes require Klf15 to form, achievement of the blood fate requires that both Ubx function be impaired and that a still unknown factor (X) be involved. In L3 larval stage, the second and third lobes are composed of heterogeneous blood progenitors. In conclusion, the mature lymph gland is composed of blood progenitors that have different origins and are generated at two different time points during development.

Our combination of lineage tracing, clonal and genetic analyses establish that each lymph gland posterior lobe originates from three embryonic pericardial cells expressing Odd and Col. These cells have bipotent blood cell/nephrocyte potential which is established during embryonic development and is later revealed at the larval stage. Bipotent blood cell/nephrocyte progenitors are similar at the L1 stage, while their progeny are heterogeneous later on in larvae, with diversity observed intra and inter posterior lobe cells. At the larval stage, Ubx and Klf15 are key regulators promoting blood cell and nephrocyte fate, respectively. This study establishes a novel paradigm, in which the acquisition of a hematopoietic progenitor fate is coupled to bipotent blood cell/nephrocyte progenitors. Our data further emphasize the sequential production of blood cell progenitors during larval development, with a developmental window controling blood cell production. This analysis reveals that genesis of the lymph gland requires two routes that are used at two different time points during development (Figure 6). In conclusion, this work provides several novel insights into the acquisition of blood cell fate during development.

At the end of embryogenesis about 110–130 pericardial cells are present, whereas in third instar larvae only 38–40 nephrocytes remain, indicating that most embryonic pericardial cells disappear during larval development (Rizki and Rizki, 1978; Ward and Coulter, 2000; Sellin et al., 2006; Monier et al., 2007; Bataille et al., 2015). In each embryonic abdominal hemi-segment, there are ten pericardial cells classified into four different types, based on the expression of various transcription factors. In this study we establish that the surviving pericardial cells are the four Odd^+^Col^+^ positive cell subsets. In segments A1 and A2, the anterior-most cell of these 4 cells gives rise to one larval nephrocyte, and the three others make up one lymph gland posterior lobe. At the end of L1 stage, while polyploidisation occurs in future nephrocytes, the pericardial cells fated to form lymph gland hematopoietic cells enter proliferation to give rise to posterior lobes. One question that remains is how some pericardial cells in A1 and A2 segments escape polyploidisation, which is common to many larval tissues, including other surviving pericardial cells.

Analyzing the *Klf15* mutant phenotype confirms that Klf15 is required for larval nephrocyte formation (Ivy et al., 2015) and further establishes that embryonic pericardial cells in the posterior aorta (A1-A4 segments) have bipotent blood cell/nephrocyte potential. Indeed, ectopic small posterior lobes are generated at the expense of nephrocytes, indicating that all embryonic pericardial cells in the posterior aorta have the potential to become hematopoietic cells. However, *Klf15* overexpression in embryonic pericardial cells does not prevent posterior lobe formation, establishing that *Klf15* is required but not sufficient to confer nephrocyte identity to bipotent blood cell/nephrocyte progenitors.

Since Ubx is the only homeotic gene expressed in A1-A2 segments in the dorsal vessel (Bataille et al., 2015), we wondered whether Ubx plays a role in lymph gland posterior lobe formation in larvae. We found that Ubx function has to be inhibited at the larval stage to permit blood cell fate (Figure 6). Published data indicate that in early embryos, Ubx is required to repress blood fate in A1-A4 abdominal segments (Mandal et al., 2004; Perrin et al., 2004). Altogether these data reveal a key role for Ubx during embryonic and larval development in regulating blood cell fate. However, even if bipotent blood/nephrocyte progenitors are present in A1-A4 segments, posterior lobes form only in A1-A2 segments, indicating that additional information, still unknown, restricts blood fate potential to only these two segments. Identifying genes involved in promoting (in A1-A2) or repressing blood fate (in A3-A4) deserves additional investigation.

In L3 larvae, posterior lobe progenitors are heterogeneous with a diversity observed intra- and inter-lobe (Kanwal et al., 2021; Rodrigues et al., 2021). While at the L1 stage bipotent blood nephrocytes are very similar, the diversity in their progeny is established during larval development. Previous analysis established the lineage giving rise to Odd^+^ embryonic pericardial cells. Four Odd^+^ embryonic pericardial cells, present per hemi-segment, derive from three progenitors. Two progenitors divide asymmetrically, with each producing one Odd pericardial cell and one cardioblast. The remaining two Odd pericardial cells arise from the division of a single Odd pericardial cell progenitor (Ward and Skeath, 2000). In summary, embryonic Odd^+^ pericardial cells that give rise to a posterior lobe at larval stage have different origins. This mixed origin might contribute to generating the blood cell diversity observed inside third instar larval posterior lobes. However, since hematopoietic progenitors composing the second and third lobes are different, additional, intrinsic and/or extrinsic, information may be involved. A recent study establishes that posterior lobe progenitors are maintained by a local signaling center present in the third lobe identified via Ubx and Col expression (Kanwal et al., 2021). Finally, whether heterogeneity in posterior lobe progenitors confers specific properties on them at the pupal or adult stage, remains to be addressed.

The mature lymph gland in L3 larvae is composed of one pair of first lobes generated during embryogenesis, and two pairs of posterior lobes formed during larval development. The lymph gland is therefore composed of lobes with different segmental origins, and generated at two different time during development (Figure 6). Indeed, anterior lobes of the lymph gland form in embryos. The anterior lymph gland cells derive from the division of hemangioblasts (Mandal et al., 2004). One hemangioblast daughter cell differentiates into a cardioblast, and the other contributes to the formation of the lymph gland first lobe. In this study, we establish that the lymph gland posterior lobes originate from Odd^+^Col^+^ bipotent blood cell/nephrocyte precursors. In L3 larvae, the lymph gland first lobe pair contains both progenitors and differentiating blood cells, while the posterior lobe cells do not show signs of differentiation up to dispersion of the lymph gland at metamorphosis (Kanwal et al., 2021; Rodrigues et al., 2021). The sequential formation of primary versus posterior lymph gland lobes and the diversity of posterior lobe cells raises the question of whether blood progenitors issued from the first lobe and posterior lobes share similar potential, or whether they have specific properties/functions in adults.

## Data Availability

The original contributions presented in the study are included in the article/Supplementary Material, further inquiries can be directed to the corresponding authors.

## Supplementary Material

Supplementary Figure S1 Expression profile of the *76E11*-Gal4 driver and lineage tracing of embryonic pericardial cells using *76E11, prc and eve* Gal4 drivers **(A,A’)** Lymph glands of mid L3 larvae (ML3) and G-Trace experiments performed with prc-Gal4 (prc>G-Trace,) and **(A’)** is an enlargement. **(A)** Col in blue, RedStinger (red), GFP (green) and Dapi (white). **(A’)** only GFP staining (green) is shown. GFP is expressed in posterior lobe cells and pericardial cells (PCs), indicating that Prc+ embryonic pericardial cells give rise to posterior lobe cells and to larval nephrocytes. **(B,B’)** Mid L3 larvae (ML3) lymph glands and lineage experiments performed with the *prc-gal4* driver. Col in red **(A)**, lineage traced cells expressing GFP in green and nuclei labelled with Dapi (blue). **(C)** Embryos at stage 16 expressing LacZ (black) with the *76E11-Gal4* driver. **(D)** Schematic representation of the four different types of pericardial cells present per embryonic abdominal hemi-segment and expressing different transcription factors. **(E,E’)** Lymph glands of mid L3 larvae (ML3) and G-Trace experiments performed with the *76E11-Gal4* driver (*76E11>G-Trace*) and nuclei labelled with Dapi (blue). **(F,F’)** Mid L3 larvae (ML3) lymph glands. G-Trace experiments performed with *eme-Gal4 (eve>G-Trace).* **(F)** Col in blue, RedStinger (red), GFP (green) and Topro (white). **(F’)** only GFP staining (green) is shown. GFP is not expressed in posterior lobe cells, indicating that Eve embryonic pericardial cells do not give rise to posterior lobe cells or to larval nephrocytes.

Supplementary Figure S2
*Klf15* is required but not sufficient for larval pericardial cell formation **(A–C’)** ML3 larvae expressing GFP (green) using the *odd-Gal4* driver, in control [*odd>GFP*, **(A,A’)**], *Klf15* knockdown [odd>RNAi-Klf15, **(B,B’)**] and Klf15 overexpression [odd>Klf15, **(C,C’)**]. *In Klf15* knockdown **(B,B’)**, PC1-3 (double arrowheads) are abnormal and resemble small posterior lobes. When *Klf15* is over-expressed in pericardial cells **(C, C’)**, no difference compared to the control **(A)** is observed.
