# Supplementary figures and images for: Identification of Bipotential Blood Cell/Nephrocyte Progenitors in Drosophila: Another Route for Generating Blood Progenitors
Source: Front Cell Dev Biol. 2022 Feb 14;10:834720. doi: 10.3389/fcell.2022.834720 (PMC8883574; doi:10.3389/fcell.2022.834720)

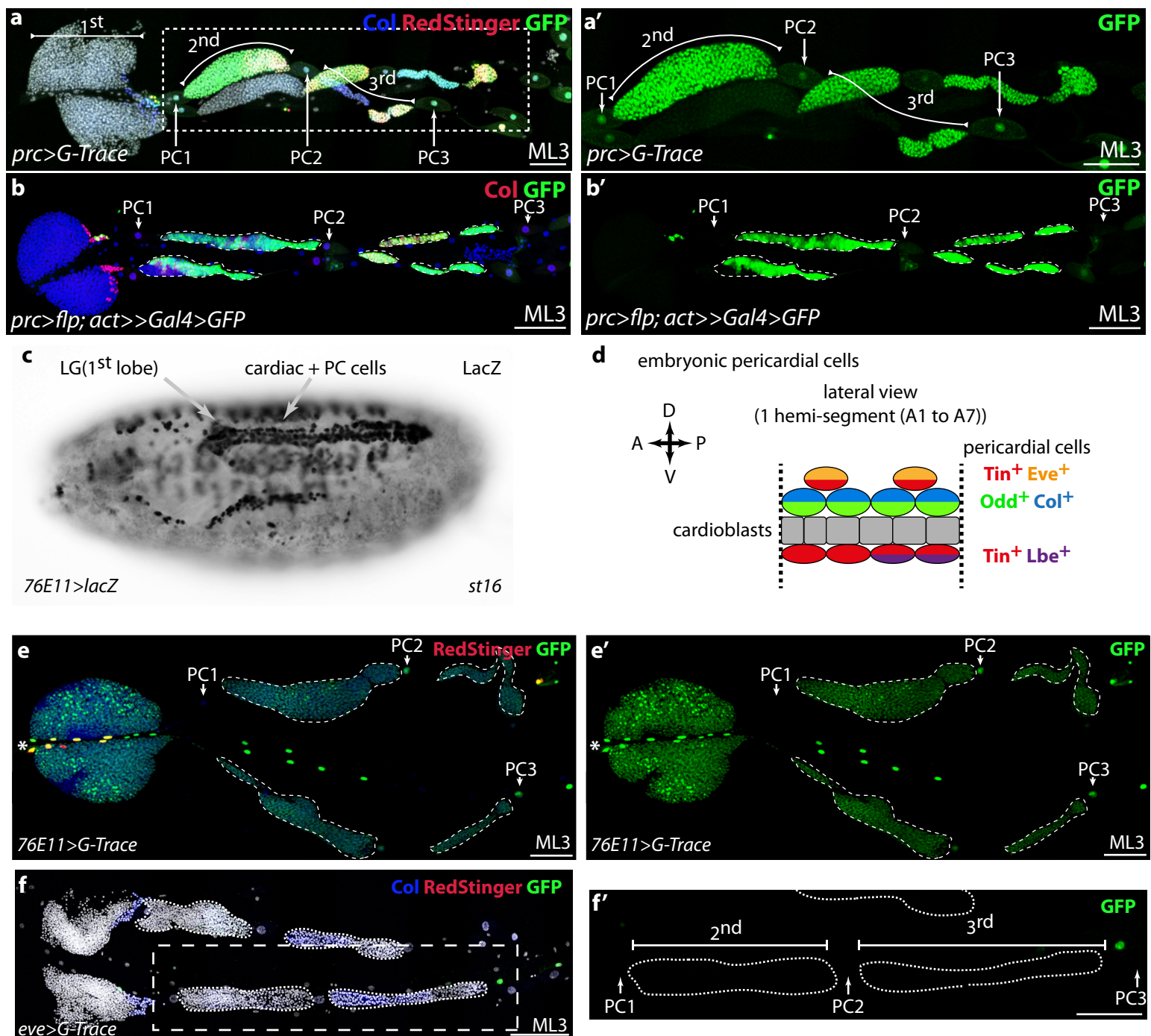

Supplementary Figure S1

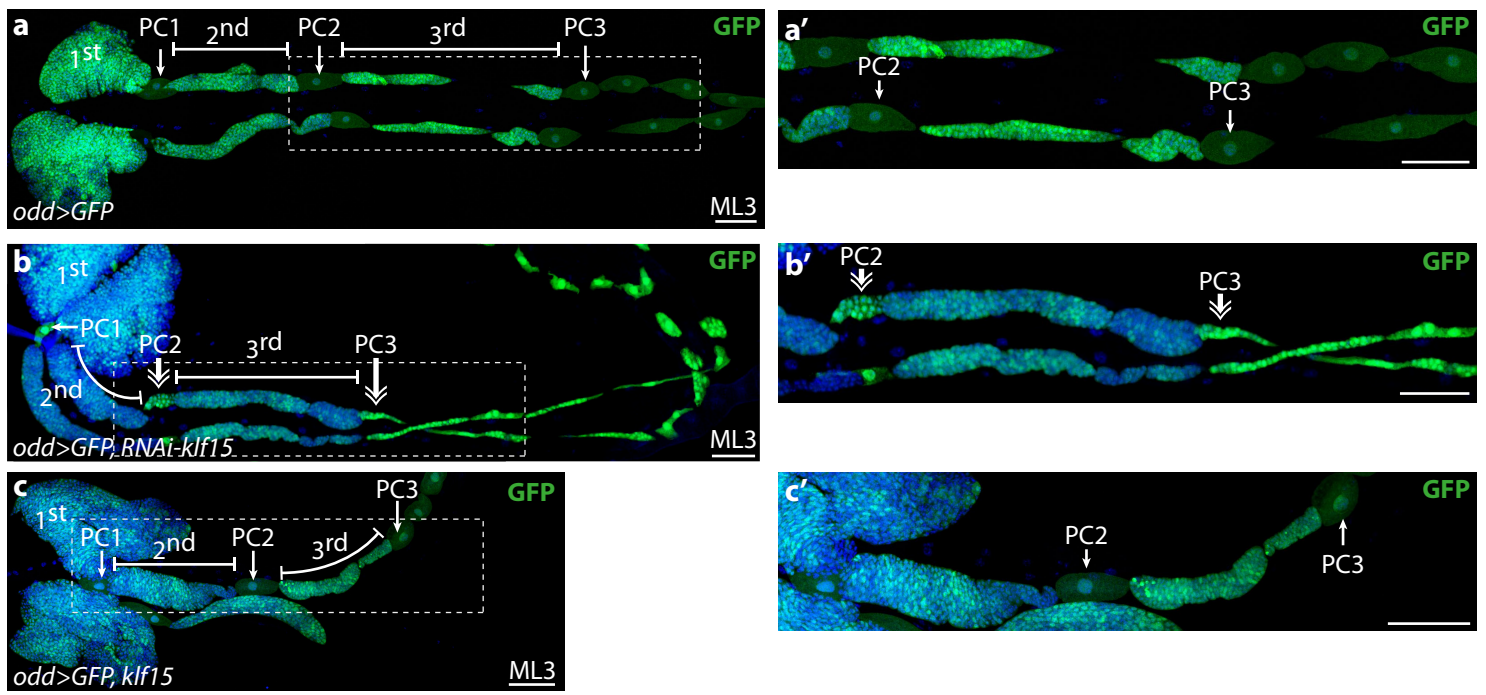

**Supplementary figure S2**

Supplement: Supplementary file 1 [file DataSheet1.pdf]
